# ﻿Two novel species of arctic-alpine lichen-forming fungi (Ascomycota, Megasporaceae) from the Deosai Plains, Pakistan

**DOI:** 10.3897/mycokeys.102.113310

**Published:** 2024-03-01

**Authors:** Muhammad Usman, Paul S. Dyer, Matthias Brock, Christopher M. Wade, Abdul Nasir Khalid

**Affiliations:** 1 Fungal Biology and Systematics Research Laboratory, Institute of Botany, University of the Punjab, Quaid-e-Azam Campus 54590, Lahore, Pakistan University of the Punjab Lahore Pakistan; 2 School of Life Sciences, University of Nottingham, Nottingham NG7 2RD, UK University of Nottingham Nottingham United Kingdom

**Keywords:** *Aspicilia*, Gilgit-Baltistan, Himalaya, Karakorum, Maximum Likelihood, Pertusariales, Skardu

## Abstract

Members of the lichen-forming fungal genus *Oxneriaria* are known to occur in cold polar and high altitudinal environments. Two new species, *Oxneriariacrittendenii* and *O.deosaiensis*, are now described from the high altitude Deosai Plains, Pakistan, based on phenotypic, multigene phylogenetic and chemical evidence. Phenotypically, *O.crittendenii* is characterised by orbicular light-brown thalli 1.5–5 cm across, spot tests (K, C, KC) negative, apothecia pruinose, hymenium initially blue then dark orange in response to Lugol’s solution. *Oxneriariadeosaiensis* is characterised by irregular areolate grey thalli 1.5–2 cm across, K test (light brown), KC test (dark brown), apothecia epruinose, hymenium initially blue then dark blue in response to Lugol’s solution. Both species share the same characters of thalli with black margins and polarilocular ascospores. The closest previously reported species, *O.pruinosa*, differs from *O.crittendenii* and *O.deosaiensis* in having non-lobate margins, thin thalline exciple (45–80 μm thick), short asci (55–80 × 25–42 μm) and K positive (yellow) and KC negative tests and divergent DNA sequence in the ITS, LSU and mtSSU regions. The newly-described *Oxneriaria* species add to growing evidence of the Deosai Plains as a region of important arctic-alpine biodiversity.

## ﻿Introduction

The Deosai Plains are located between the Himalaya and Karakorum, two of the world’s most famous mountain ranges, with an average elevation of over 4,000 m (Woods et al. 1997). They represent one of the most important high altitude alpine grasslands and summer pastures of the trans-Himalayan range in Pakistan. Three important river systems originate from the Deosai Plains, namely the Shatung, Bara Pani and Kala Pani, which combine to form the Shigar River, an important tributary of the Indus River (Hussain 2014). The Deosai Plains are characterised by an undulating topography with a range of edaphic conditions and ecological niches present that are subject to extreme cold conditions for long periods of the year. A diverse range of flora and fauna have been recorded, which are considered to be adapted for survival under such conditions (Woods et al. 1997; Usman et al. 2021). The highland arctic-alpine ecosystem includes herbaceous perennial grasses and sedges which dominate the vegetation of the plateau, forming dense moist grasslands in the valley plains, whilst dwarfed and stunted vegetation, flower fields, rocky outcrops and soil crusts are also present (Stewart 1961; Hussain et al. 2015).

Members of the lichen-forming genus *Oxneriaria* S.Y. Kondr. & Lőkös are distributed in cold polar and high-altitude localities of Eurasia and the Northern Hemisphere (Nordin et al. 2011; Haji-Moniri et al. 2017; Chesnokov et al. 2018; Halıcı et al. 2018). They are characterised by the presence of a radiating lichen thallus with a wrinkled or lobate peripheral zone, relatively small ascospores, production of substictic acid and positioning as a distinct branch on phylogenetic trees in the Megasporaceae. They grow on rocks and have been observed growing side by side with other taxa of the same and other genera (Haji-Moniri et al. 2017). The genus was first named by Haji-Moniri et al. (2017) who transferred over nine species that were previously included in the genus *Aspicilia*. A total of fourteen species have, so far, been described for the genus *Oxneriaria* (Haji-Moniri et al. 2017; Asghar et al. 2023; Iqbal et al. 2023; Zulfiqar et al. 2023).

Four species of the genus *Oxneriaria* have, so far, been described from Pakistan with a distance of 300 to 650 km from Deosai Plains, namely *O.iqbalii* R. Zulfiqar, H. S. Asghar, K. Habib & Khalid from Kohistan (350 km) and Swat (500 km), *O.kohistaniensis* R. Zulfiqar, K. Habib & Khalid from Kohistan (350 km), *O.pakistanica* M. S. Iqbal, Usman, K. Habib & Khalid from Darel (300 km) and *O.pruinosa* H. S. Asghar., Usman, K. Habib & Khalid from Chitral (650 km). These were all found at relatively high altitudes up to ca. 2,500 m (Asghar et al. 2023; Iqbal et al. 2023; Zulfiqar et al. 2023). During the period 2019 to 2020, several collections of lichens were made from the Deosai Plains and adjacent localities at altitudes above 4,000 m. From this collection, four samples were attributed to the genus *Oxneriaria*, which comprised two new species as will be described in this study.

## ﻿Materials and methods

### ﻿Sample collection

More than half of the Deosai Plains are situated between an elevation (elev.) of 4,000 and 4,500 m with an average daily temperature ranging from -20 °C (January-February) to 12 °C (July-August). Annual precipitation varies from 350 to 550 mm, mostly received during winter as snow (WAPDA 2012; Usman et al. 2021). Lichen collections were made from both rock and soil crusts during the period May 2019 to Sept 2020 from various locations in the Deosai Plains National Park, Gilgit Baltistan, Pakistan (see later for precise collection site details for particular specimens) at altitudes between 4,177 and 4,689 m. Samples were air dried before storage and examination.

### ﻿Morpho-anatomical and chemical studies

Methods for the examination of external morphology, macroscopic and microscopic characters and their measurements were followed and recorded according to the terminology of Ryan et al. (2002). All the measurements of anatomical structures were noted in water with an average of 25 ascospores per collection and 5 - 6 sections were prepared for the thallus, apothecia and pycnidia. The algal partner was identified by following Friedl and Büdel (2008). For thallus chemical reactions, standard K (5% potassium hydroxide aqueous solution), C (commercial bleach), KC (commercial bleach after 5% potassium hydroxide aqueous solution) and ultra-violet (UV) tests were done. Solvents A (toluene/dioxane/ acetic acid as 180:45:5) and G (toluene/ ethyl acetate/ formic acid as 139:83:8) were used for the detection of secondary metabolites through thin layer chromatography (TLC) as described by Orange et al. (2010).

### ﻿Molecular and phylogenetic analyses

Nuclear DNA was extracted from apothecia present on thalli using a GF1 Plant DNA extraction kit according to the manufacturer’s instruction (Vivantis, Selangor Darul Ehsan, Malaysia). Primers used for amplifications were ITS1F 5'-CCT GGT CAT TTA GAG GAA GT A A-3 ' and ITS4 5'-TCC TCC GCT CTA TTG ATA TGC-3' for the internal transcribed spacer (ITS1-5.8S-ITS2) region, while LROR 5'-ACC CGC TGA ACT TAA GC-3' and LR5 5'-TCC TGA GGG AAA CTT CG-3' were used for the nuclear large subunit (LSU) ribosomal RNA region (White et al. 1990; Gardes and Bruns 1993). For the mitochondrial (mt) small subunit (SSU) ribosomal RNA region, SSU1 5'-AGC AGT GAG GAA TAT TGG TC-3' and SSU3R 5'-ATG TGG CAC GTC TAT AGC CC-3' were used (Zoller et al. 1999). Polymerase chain reaction (PCR) conditions adapted from those of Gardes and Bruns (1993) were followed according to Zoller et al. (1999) and Usman and Khalid (2020). The PCR amplicons were purified using a QIAquick PCR Purification Kit (Qiagen, Valencia, CA, USA) and then sent for sequencing to TsingKe, China.

Forward and reverse sequences of the ITS, LSU and mtSSU regions were obtained in FASTA format and sequences were assembled using BIOEDIT v. 7.2.5 (Hall 1999). These were compared with related DNA sequences available online through BLAST at NCBI (https://www.ncbi.nlm.nih.gov/guide). The sequences used in the ITS, LSU and mtSSU dataset were retrieved from the NCBI database, based on similarity of 93% identity or greater, plus all published sequences from the genus *Oxneriaria* (Nordin et al. 2007; Nordin et al. 2011; Asghar et al. 2023; Iqbal et al. 2023; Zulfiqar et al. 2023). Sequences of *Megasporacretacea* Gasparyan, Zakeri & Aptroot were used as an outgroup in the ITS phylogenetic tree, while *Megasporaverrucosa* (Ach.) Arcadia & A. Nordin was used as outgroup in the LSU and mtSSU phylogenetic trees (Nordin et al. 2010; Sohrabi et al. 2013; Zakeri et al. 2016). Sequences used for the phylogenetic analyses are presented in Table 1 together with GenBank accession numbers, voucher numbers and country distribution. The final alignments of sequences were made in SEAVIEW software version 5.0.5 using the CLUSTAL W method (Gouy et al. 2010). Maximum Likelihood phylogenetic trees were inferred in RAxML-HPC2 using XSEDE (8.2.10) using the GTR+GAMMMA nucleotide substitution model and with 1000 bootstrap replicates. Phylogenetic analyses were undertaken using the CIPRES online portal (https://www.phylo.org/), with substitution model verified using jModelTest 2.1.6 and the Akaike Information Criterion (Akaike 1974; Darriba et al. 2012) to determine the best nucleotide substitution model. Phylogenetic trees were visualised using FigTree v. 1.4.2 (Rambaut 2012). Newly-generated sequences were deposited in GenBank (accession numbers OR037219–OR037226, OR037259–OR037262, Table 1). These were investigated further by DNA-based phylogenetic analyses and detailed morpho-anatomical and chemical studies as follows.

**Table 1.** Sequences used in the phylogenetic analyses. Novel sequences generated during this study are shown in bold. Note that sequences were not available for all regions for certain taxa.

Taxon name	Voucher number	GenBank accession			Country
		ITS	LSU	mt SSU	
***Oxneriariacrittendenii***	**LAH3719**3	**OR037223**	OR037219	**OR037259**	**Pakistan**
***Oxneriariacrittendenii***	**LAH37194**	**OR037224**	**OR037220**	**OR037260**	**Pakistan**
*Oxneriariadendroplaca*	UPS:Nordin 5952	HQ259259	HM060744	HM060706	Sweden
*Oxneriariadendroplaca*	UPS:Nordin 6366	HQ259260	HM060758	–	Finland
***Oxneriariadeosaiensis***	**LAH37200**	**OR037225**	**OR037221**	**OR037261**	**Pakistan**
***Oxneriariadeosaiensis***	**LAH37416**	**OR037226**	**OR037222**	**OR037262**	**Pakistan**
*Oxneriariaiqbalii*	LAH37155	ON392710	–		Pakistan
*Oxneriariaiqbalii*	LAH37156	ON392709	ON392708		Pakistan
*Oxneriariakohistaniensis*	LAH37152	ON392707	ON392711	–	Pakistan
*Oxneriariakohistaniensis*	LAH37151	ON454505	–	–	Pakistan
*Oxneriariamashiginensis*	Nordin 5790 (UPS)	EU057912	HM060732	HM060694	Sweden
*Oxneriariamashiginensis*	UPS:Tibell 23557	HQ259266	–	–	Sweden
*Oxneriariapakistanica*	LAH37495	OP114649	–	–	Pakistan
*Oxneriariapakistanica*	LAH37501	OP627196	–	–	Pakistan
*Oxneriariapermutata*	Nordin 6027 (UPS)	EU057918	HM060747	HM060709	Sweden
*Oxneriariapermutata*	Nordin 6029 (UPS)	EU057919	–	–	Sweden
*Oxneriariapermutata*	Nordin 6039 (UPS)	EU057921	–	–	Sweden
*Oxneriariapermutata*	Nordin 5980 (UPS)	EU057930	–	–	Sweden
*Oxneriariapermutata*	Wheeler 4463	–	–	MW424810	Alaska, USA
*Oxneriariapruinosa*	LAH37556	OP352770	–	–	Pakistan
*Oxneriariapruinosa*	LAH37555	OP352771	–	–	Pakistan
*Oxneriariarivulicola*	Nordin 5957 (UPS)	EU057922	HM060753	–	Sweden
*Oxneriariarivulicola*	Nordin 5960 (UPS)	EU057923	–	–	Sweden
*Oxneriaria* sp	Nordin 6003 (UPS)	EU057931	–	–	Sweden
*Oxneriaria* sp	Nordin 6004 (UPS)	EU057932	–	–	Sweden
*Oxneriariasupertegens*	Owe-Larsson H-168a (UPS)	EU057935	–	–	Sweden
*Oxneriariasupertegens*	Owe-Larsson 9011 (UPS)	EU057937	–	–	Norway
*Oxneriariasupertegens*	Nordin 6023 (UPS)	EU057938	HM060751	–	Sweden
*Oxneriariasupertegens*	Owe-Larsson 9002 (UPS)	–	HM060742	HM060704	Norway
*Oxneriariaverruculosa*	Owe-Larsson 9007 (UPS)	EU057940	HM060741	HM060703	Norway
*Oxneriariaverruculosa*	Owe-Larsson 9003 (UPS)	EU057941	–	–	Norway
*Oxneriariaverruculosa*	Nordin 5942 (UPS)	EU057942	–	–	Sweden
*Oxneriariavirginea*	UPS:Nordin 6017a	HQ259270	–	–	Sweden
*Oxneriariavirginea*	UPS:Ebbestad SVL1-1	HQ259271	–	–	Svalbard
*Oxneriariavirginea*	Wheeler 7153 (hb. Wheeler)	–	–	MW424818	Montana, USA
**Outgroup**					
*Megasporacretacea*	B 600200932	KX253974	–	–	Armenia
*Megasporacretacea*	B 600199170	KX253975	–	–	Armenia
*Megasporaverrucosa*	St. Clair C54042 (BRY)	–	KC667062	–	Colorado, USA
*Megasporaverrucosa*	UPS:Nordin 6495	–	–	HM060687	Sweden

## ﻿Results

**Figure 1.**
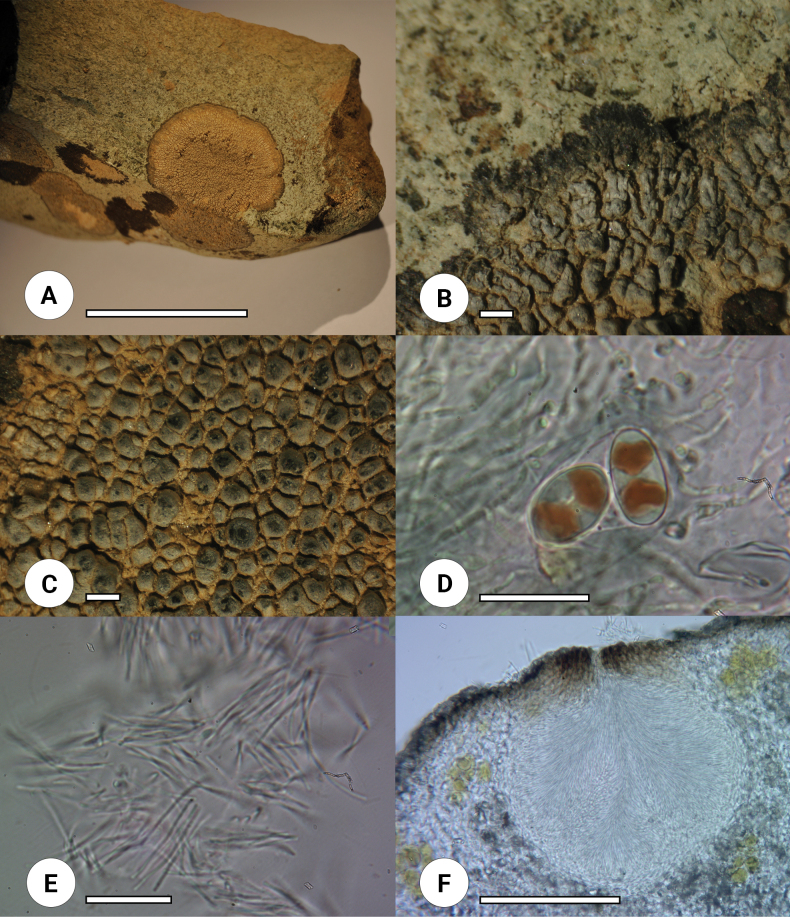
*Oxneriariacrittendenii* sp. nov. holotype (LAH37193) **A** thallus **B** margins **C** apothecia under stereomicroscope **D** ascospores in Lugol’s solution **E** conidia **F** pycnidium. Photos by Muhammad Usman. Scale bars: 5 cm (**A**); 1 mm (**B, C**); 20 μm (**D, E**); 100 μm (**F**).

**Figure 2.**
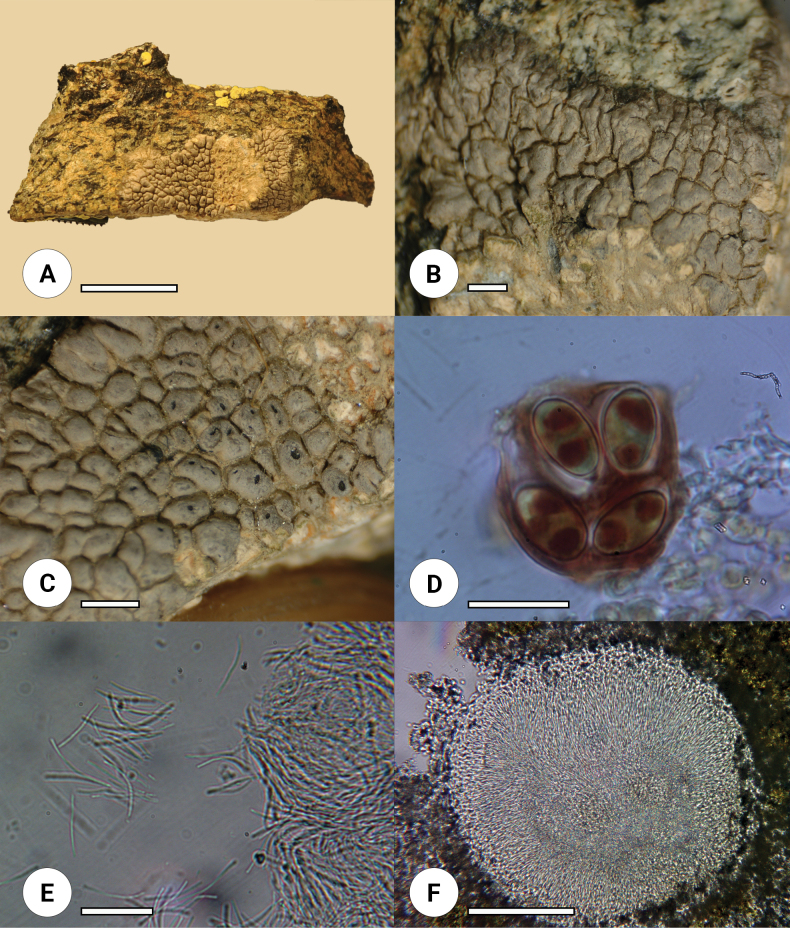
*Oxneriariadeosaiensis* sp. nov. holotype (LAH37200) **A** thallus **B** margins **C** apothecia under stereomicroscope **D** ascospores in Lugol’s solution **E** conidia **F** pycnidium. Photos by Muhammad Usman. Scale bars: 1 cm (**A**); 1 mm (**B, C**); 20 μm (**D**); 30 μm (**E**); 100 μm (**F**).

Out of almost 300 samples collected from the Deosai plains and its adjacent areas during the 2019 and 2020 surveys, four lichen thalli were putatively assigned to the genus *Oxneriaria* on the basis of gross morphological features (Figs 1, 2).

### ﻿Multigene phylogenetic analyses

DNA was extracted from the four different collections and used successfully in PCR to generate amplicons for the ITS, LSU and mtSSU regions, which ranged in size from 500–800, 900–950 and 900–960 base pairs, respectively. Sequence data of amplicons were aligned and used to construct separate ITS, LSU and mtSSU trees via Maximum Likelihood analyses to examine phylogenetic relationships. Distinct, well-supported clades were recovered from all datasets with minimal conflict, each taxon showing a unique position in all phylogenetic analyses with sequence divergence from other taxa. Clade names were provisionally assigned.

**Figure 3.**
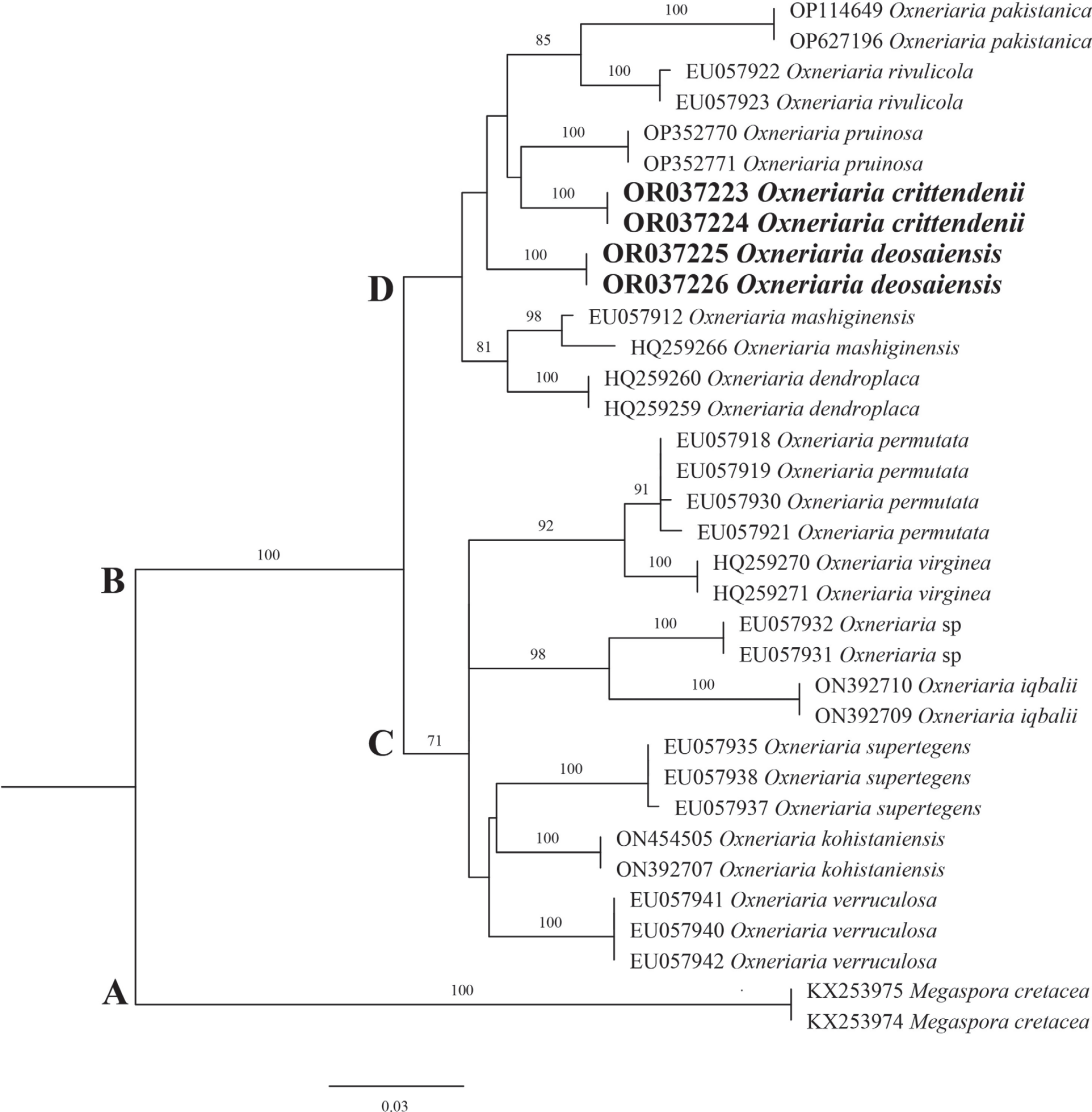
Phylogenetic tree of the genus *Oxneriaria* as generated by Maximum Likelihood (ML) analyses, based on ITS sequences. Bootstrap values > 70%, based on 1,000 replicates are shown at the branches. Novel sequences, generated during this study, are shown in bold.

The ITS phylogenetic tree (Fig. 3) consisted of sequences from a total of 34 taxa including the outgroup clade A comprised of two sequences of *Megasporacretacea* (KX253975, KX253974) and 32 sequences representing an *Oxneriaria* ingroup (Clade B), which could be further subdivided into two main clades C and D. Clade D consisted of a total of seven species of *Oxneriaria* including new, well-supported sequences named here *O.crittendenii* and *O.deosaiensis*, each represented by two of the four field collections. Within clade D, *Oxneriariadeosaiensis* formed a separate branch, sister to a clade which consisted of four species, namely *O.crittendenii*, *O.pakistanica*, *O.pruinosa* and *O.rivulicola* (H. Magn.) S. Y. Kondr. et L. Lőkös and showed 5%, 7.2%, 5.1% and 5% bp differences with *O.deosaiensis* in the sequences of ITS region, respectively, whilst *O.crittendenii* showed 6.1%, 5.3% and 5% bp differences with *O.pakistanica*, *O.pruinosa* and *O.rivulicola*, respectively. The closest species to *O.crittendenii* and *O.deosaiensis* was *O.pruinosa*, forming a separate branch.

**Figure 4.**
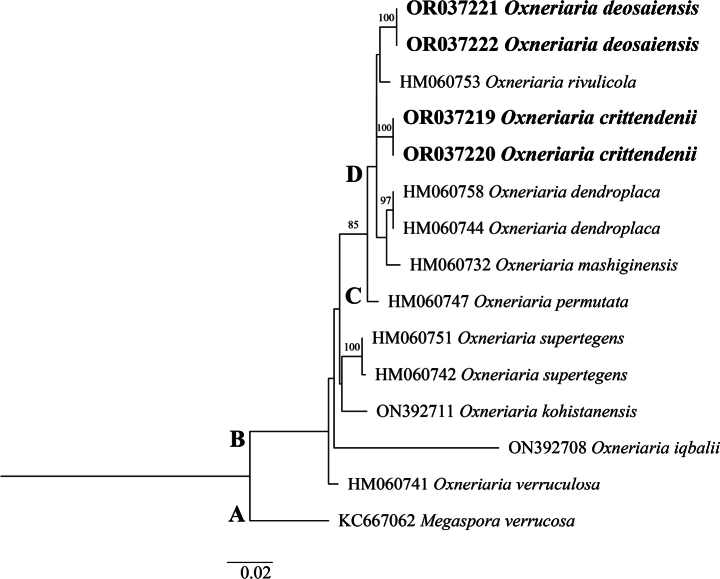
Phylogenetic tree of the genus *Oxneriaria* as generated by Maximum Likelihood (ML) analyses, based on LSU sequences. Bootstrap values > 70%, based on 1,000 replicates are shown at the branches. Novel sequences, generated during this study, are shown in bold.

The LSU phylogenetic tree (Fig. 4) similarly revealed that *O.crittendenii* and *O.deosaiensis* are positioned on well-supported branches and are monophyletic. The tree consisted of a total 15 available sequences of which 14 sequences represent an *Oxneriaria* ingroup (Clade B), while *Megasporaverrucosa* (Ach.) Arcadia & A. Nordin (KC667062) formed an outgroup (Clade A). Clade B could be further subdivided into Clades C and D. *Oxneriariadeosaiensis*, *O.crittendenii*, *O.dendroplaca* (H. Magn.) S. Y. Kondr. et L. Lőkös., *O.rivulicola* and *O.mashiginensis* (Zahlbr.) S. Y. Kondr. et L. Lőkös. were all positioned in the same clade (Clade D), where they each formed separate branches. Sequences of *O.deosaiensis* for the LSU region showed 1.2%, 1.5%, 1.2% and 2%, bp differences to *O.crittendenii*, *O.dendroplaca*, *O.rivulicola* and *O.mashiginensis*, respectively, whilst *Oxneriariacrittendenii* showed 1.5%, 1.4%, 1.5 and 2% bp differences with *O.rivulicola*, *O.dendroplaca* and *O.mashiginensis*, respectively.

**Figure 5.**
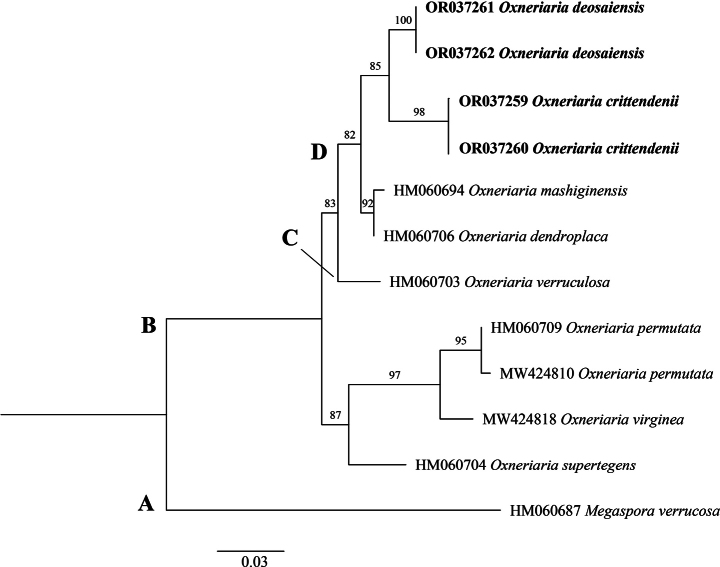
Phylogenetic tree of the genus *Oxneriaria* as generated by Maximum Likelihood (ML) analyses, based on mtSSU sequences. Bootstrap values > 70%, based on 1,000 replicates are shown at the branches. Novel sequences, generated during this study, are shown in bold.

The mtSSU phylogenetic tree (Fig. 5) consisted of a total 12 available sequences of which 11 sequences represented an *Oxneriaria* ingroup (Clade B), while *Megasporaverrucosa* (HM060087) was used as an outgroup (Clade A). *Oxneriariadeosaiensis*, *O.crittendenii*, *O.dendroplaca* and *O.mashiginensis* formed a clade (Clade D) distinct from *O.verruculosa* forming clade C. Sequences of *O.deosaiensis* from the mtSSU region showed 1%, 2% and 2.5% bp differences with the sequences of closest species *O.crittendenii*, *O.dendroplaca* and *O.mashiginensis*, respectively, whilst the *O.crittendenii* showed 2.1% and 2.4% bp differences with *O.dendroplaca* and *O.mashiginensis*, respectively. Thus, the mtSSU analysis again showed that sequences of *O.crittendenii* and *O.deosaiensis* are positioned on well-supported branches.

### ﻿Taxonomy

#### ﻿ Oxneriaria crittendenii

Taxon classificationAnimaliaPertusarialesMegasporaceae

Usman & Khalid

AB0F169D-D710-5ACF-848B-67A78DEAE60E

848889

Fig. 1

##### Etymology.

The specific epithet “*crittendenii*” refers to the British lichenologist Prof. Peter D Crittenden in recognition for his outstanding contributions to lichenology.

##### Holotype.

Pakistan. Gilgit Baltistan: Deosai Plains (35°0'45.73"N, 75°13'25.95"E, elev. 4,651 m) on rocks, 13 May 2019, M. Usman DEO117 (LAH, holotype; LAH37193). GenBank OR037223 [ITS], OR037219 [LSU], OR037259 [mtSSU].

##### Diagnosis.

It differs from its closest species *O.pruinosa* by having lobate black margins (vs. non-lobate), orbicular thallus 1.5–5 cm (vs. irregular 3–8 cm), K test negative (vs. K positive yellow), distinct proper-exciple 17–40 µm wide (vs. indistinct) and polarilocular ellipsoid ascospores (vs. simple ellipsoid).

##### Description.

Thallus crustose, epilithic, orbicular, 1.5–5 cm across, zonate, fine bullate to areolate in the centre to poorly areolate towards margin, in the centre areoles 0.5–1 mm diam. and a few areoles changing to squamules up to 1.8 mm in length, lobate at margins, determinate and radiate. ***Hypothallus*** distinct, shiny light brown. ***Upper surface*** grey with white powdery texture and black at margins. ***Thallus*** heteromerous, upper cortex 20–60 µm thick, globose to sub-globose hyaline paraplectenchymatous cells, 6–11 µm in diam. ***Algal layer*** discontinuous, 90–140 µm thick, photobiont *Trebouxia* sp, coccoid cells, globose to sub-globose 6–14 µm in diam. ***Medulla*** and ***lower cortex*** not differentiated and consisting of paraplectenchymatous, globose to sub-globose hyaline cells 25–45 µm in diam.

***Apothecia*** without stipe, aspicilioid, one apothecium per areole, rounded, 600–950 µm in diam., pruinose with black disc 450–700 µm, dull and concave. ***Proper exciple*** 17–40 µm thick. ***Thalline exciple*** 140–190 µm thick. ***Epihymenium*** brown, 10–20 µm thick. ***Hymenium*** hyaline, 85–110 µm thick. ***Hypothecium*** hyaline, 35–55 µm thick. ***Asci*** clavate, 8–spored, 60–100 × 22–30 µm. ***Ascospores*** hyaline, ellipsoid, polarilocular, 13–18 × 7–11 µm. ***Paraphyses*** moniliform, septate, cylindrical cells 3–10 × 1–2.5 µm, with internally brown terminal cells. ***Pycnidia*** roccella type (Ryan et al. 2002), globose to pyriform, 115–200 × 85–200 µm dark brown ostiole, long filiform hyaline conidia, 17–24 × 1 µm.

##### Ecology.

Saxicolous, calcareous, known only from Deosai Plains, Gilgit-Baltistan, occurring at elevations between 4,117 m and 4,651 m in extremely cold conditions.

##### Chemical study:

K -ve, C -ve, KC -ve, UV +ve (light green), hymenium initially blue then turning dark orange after Lugol’s solution. Substictic acid detected through TLC.

##### Additional material examined.

Pakistan. GILGIT BALTISTAN: Deosai Plains, 35°7'22.48"N, 75°36'35.09"E, elev. 4,177 m, on rocks, 3 September 2020, M. Usman & M. Shafiq DEO129 (LAH, paratype; LAH37194; GenBank OR037224 [ITS], OR037220 [LSU], OR037260 [mtSSU].

#### ﻿ Oxneriaria deosaiensis

Taxon classificationAnimaliaPertusarialesMegasporaceae

Khalid & Usman

0AD8C319-B561-5C27-A494-DF2CDCBE1D26

848890

Fig. 2

##### Etymology.

The specific epithet “*deosaiensis*” refers to the Deosai Plains, the type locality.

##### Holotype.

Pakistan. Gilgit Baltistan: Deosai Plains (35°0'10.06"N, 75°15'0.45"E, elev. 4,689 m) on soil, 13 May 2019, M. Usman DEO206 (LAH, holotype; LAH37200). GenBank OR037225 [ITS], OR037221 [LSU], OR037261 [mtSSU].

##### Diagnosis.

It differs from its closest species *O.pruinosa* by having lobate black margins (vs. non-lobate), K test positive light brown (vs. K positive yellow), KC test positive dark brown (vs. KC negative), apothecia epruinose (vs. densely pruinose), distinct proper-exciple 30–50 µm wide (vs. indistinct) and polarilocular ellipsoid ascospores (vs. simple ellipsoid).

##### Description.

Thallus crustose, epilithic, irregular, 1.5–2 cm across, zonate, areolate to poorly bullate up to 0.8 mm in diam. to lobate up to 1.5 mm at margins, determinate and radiate. ***Hypothallus*** light grey. ***Upper surface*** dull grey, black at margins. ***Thallus*** heteromerous, upper cortex 20–55 µm thick, paraplectenchymatous hyaline cells 6–15 µm in diam. ***Algal layer*** discontinuous, 50–90 µm thick, photobiont *Trebouxia* sp, coccoid cells, globose to sub-globose, 13–21 µm in diam. ***Medulla*** and ***lower cortex*** not differentiated and consisting of paraplectenchymatous, globose to sub-globose hyaline cells, 5–12 µm diam.

***Apothecia*** without stipe, aspicilioid, epruinose, one apothecium per areole, rounded, 520–700 µm in diam., with black disc 350–550 µm in diam., dull, concave. ***Proper exciple***, 30–50 µm thick. ***Thalline exciple*** 90–145 µm thick. ***Epihymenium*** brown, 10–24 µm thick. ***Hymenium*** hyaline, 90–160 µm thick. ***Hypothecium*** hyaline, 50–90 µm thick. ***Asci*** clavate, 8–spored, 75–110 × 16–27 µm. ***Ascospores*** hyaline, ellipsoid, polarilocular 11–18 × 7–10 µm. ***Paraphyses*** moniliform, septate, cylindrical cells 4–10 × 1–2 µm, with internally brown terminal cells. ***Pycnidia*** roccella type (Ryan et al. 2002), globose to pyriform, 230–320 × 210–280 µm dark brown ostiole, long filiform hyaline conidia, 19–35 × 1 µm.

##### Ecology.

Saxicolous, Quartz, known only from Deosai Plains, Gilgit-Baltistan, occurring at elevations between 4,364 m and 4,689 m in extreme cold conditions.

##### Chemical study.

K +ve (light brown), C -ve, KC +ve (dark brown), UV +ve (light green), hymenium initially blue then turning dark blue after Lugol’s solution. Substictic acid and two unknown substances detected through **TLC**.

##### Additional material examined.

Pakistan. GILGIT BALTISTAN: Deosai Plains, 35°6'28.58"N, 75°44'27.37"E, 4,364 m, on rocks, 15 May 2019, M. Usman & Kamran Habib DEO666 (LAH, paratype; LAH37416; GenBank OR037226 [ITS], OR037222 [LSU], OR037262 [mtSSU].

## ﻿Discussion

The genus *Oxneriaria* was introduced by Haji-Moniri et al. (2017) and is characterised by the presence of radiating thalli with a wrinkled or lobate peripheral zone, relatively small ascospores, the possible presence of substictic acid and phylogenetic divergence from neighbouring taxa. Four species of the genus *Oxneriaria* have recently been described from Pakistan from relatively high altitude locations, namely *O.iqbalii* from Dassu and Miandam (at elev. 1,607 m and 1,800 m), *O.kohistaniensis* from Dassu and Razika Seo Valley (at elev. 1,607 m and 1,811 m), *O.pakistanica* from Darel Valley (at elev. 1,900 m and 2,000 m) and *O.pruinosa* from Chitral (at elev. 2,550 m) (Asghar et al. 2023; Iqbal et al. 2023; Zulfiqar et al. 2023). By contrast, the two proposed new species, *O.crittendenii* and *O.deosaiensis*, reported in the current study, were found occurring at very high altitude elevations between 4,117 m and 4,689 m in environments subject to periodic extremely cold conditions.

The two new proposed species share some morphological similarities to each other such as dull coloured grey to brown, areolate to bullate, heteromerous thalli with black lobate margins, a discontinuous algal layer, medulla consisting of paraplectenchymatous cells and concave black apothecia showing as light green in response to UV. However, the two species, *O.crittendenii* and *O.deosaiensis*, also exhibit differences from each other in thallus growth- pattern (orbicular vs. irregular), hypothallus appearance (shiny brown vs. light grey), algal layer (90–140 µm vs. 50–90 µm), size of algal cells (6–14 µm vs. 13–21 µm), size of lower cortex cells (25–45 µm vs. 5–12 µm), apothecia (pruinose 0.6–0.95 mm diam. vs. epruinose 0.52–0.7 mm diam.) and thalline exciple (140–190 µm vs. 90–145 μm thick) and hypothecium (35–55 µm vs. 50–90 µm thick), respectively. Additionally, in response to Lugol’s solution, the hymenium of *O.crittendenii* turned dark orange, whilst that of *O.deosaiensis* turned dark blue.

The two new proposed species *O.crittendenii* and *O.deosaiensis* were found to be phylogenetically closely related to certain other *Oxneriaria* species, in particular *O.pakistanica*, *O.pruinosa* and *O.rivulicola*, although clear molecular differences were apparent in the ITS, LSU and mtSSU sequences. There were, in addition, some striking phenotypic characters showing the distinctive characteristics of the novel taxa along with the closest species and these are shown in Table 2.

**Table 2.** Comparison of closely-related species of *Oxneriaria* with novel taxa.

Characters	*O.crittendenii*	*O.deosaiensis*	*O.pakistanica*	*O.pruinosa*	*O.rivulicola*
**Margins**	lobate, determinate, black	lobate, determinate, black	areolate, indeterminate, whitish-grey	lobate, determinate, whitish-grey	Non-elongate areoles
**Hypothallus**	shiny light brown	light grey	light brown	light grey	light grey
**Upper Cortex**	20–60 µm thick	20–55 µm thick	10–25 μm thick	30–50 μm thick	25–40 μm thick
**Algal Layer (thick)**	90–140 µm	50–90 µm	30–50 μm	70–140 μm	30–50 μm
**Algal Cells (in diam.)**	6–14 µm	13–21 µm	10–15 μm	10–17 μm	7–15 μm
**Apothecia (in diam.)**	pruinose, 450–700 µm	epruinose, 520–700 µm	epruinose, up to 2 mm	densely pruinose, up to 1 mm	up to 2 mm
**Hypothecium (thick)**	35–55 µm	50–90 µm	90–170 μm	50–120 μm	80–100 μm
**Asci**	60–100 × 22–30 µm	75–110 × 16–27 µm.	60–80 × 30–40 μm	55–80 × 25–42 μm	70–85 × 20–24 μm
**Pycnidia**	roccella type	roccella type	absent	globose	globose
**Conidia**	17–24 × 1 µm	19–35 × 1 µm	absent	14–18 × 1 µm	30–37 × 1 µm
**References**	**This Study**	**This Study**	**Iqbal et al. (2023)**	**Asgar et al. (2023)**	**Magnusson (1923); Nordin et al. (2011)**

In addition to the differences of Table 2, *O.crittendenii* and *O.deosaiensis* have polarilocular ellipsoid ascospores whilst *O.pakistanica*, *O.pruinosa* and *O.rivulicola* have simple ellipsoid ascospores. Chemically, *O.crittendenii* showed no change to K and KC tests, whilst the thalli of *O.deosaiensis* turned light brown and dark brown in response to K and KC tests, respectively. By contrast to these tests, *O.pakistanica* showed positive K (yellowish green) and KC (light green) tests, *O.pruinosa* showed K positive (yellow) and KC negative tests (Asghar et al. 2023; Iqbal et al. 2023), whilst *O.rivulicola* showed no change to K and KC tests (Magnusson 1923; Nordin et al. 2011).

## ﻿Conclusions

In summary, as a result of all the distinct phenotypic and phylogenetic characters, we here propose the addition of two new species in the genus *Oxneriaria* from high altitudinal environments in Pakistan. Whilst these were found infrequently, the detection of the two new species *O.crittendenii* and *O.deosaiensis* add to reports of the discovery of other new species of lichen-forming fungi from the Deosai Plains in Pakistan (Usman et al. 2021, 2023), emphasising the importance of this region as a site of arctic-alpine biodiversity.

## Supplementary Material

XML Treatment for Oxneriaria crittendenii

XML Treatment for Oxneriaria deosaiensis

## ﻿Additional information

### Author ORCIDs

Muhammad Usman https://orcid.org/0000-0002-3490-058X

Paul S. Dyer https://orcid.org/0000-0003-0237-026X

Matthias Brock https://orcid.org/0000-0003-2774-1856

Christopher M. Wade https://orcid.org/0000-0002-1269-9694

Abdul Nasir Khalid https://orcid.org/0000-0002-5635-8031

### Data availability

All of the data that support the findings of this study are available in the main text.
